# Toward Long-Term Sailing Robots: State of the Art From Energy Perspectives

**DOI:** 10.3389/frobt.2021.787253

**Published:** 2022-01-05

**Authors:** Qinbo Sun, Weimin Qi, Hengli Liu, Xiaoqiang Ji, Huihuan Qian

**Affiliations:** ^1^ Shenzhen Institute of Artificial Intelligence and Robotics for Society (AIRS), The Chinese University of Hong Kong, Shenzhen, China; ^2^ School of Science and Engineering, The Chinese University of Hong Kong, Shenzhen, China

**Keywords:** autonomous sailing robots, actuation in sailing, energy harvesting, energy management, energy sustainability

## Abstract

Sailing robots can contribute significantly to maritime surface exploration, due to its potential for long-range and long-duration motions in the environment with abundant wind. However, energy, the critical factor for their long-term missions, shall be carefully investigated, so as to achieve sustainability in distance and time. In this survey, we have conducted a comprehensive investigation on numerous sailing robots, developed in academia and industry. Some of them have achieved long-term operation, and some are motivated by, but still on the way to this ambitious goal. Prototypes are grouped in each team, so as to view the development path. We further investigate the existing design and control strategies for energy sufficiency from three perspectives: actuation, harvesting, and energy management. In propulsion and steering, i.e., two major actuations, researchers have accumulated effective sail and rudder designs. The motorized propeller and wave-glider–inspired mechanism also contribute as compliments for propulsion. Electricity harvesting based on solar or wind energies is also discussed to gather more power from nature. Pros and cons in strategies of energy management, which are valuable tools to enhance power utilization efficiency, are elaborated. This article is hoped to provide researchers in long-term robotic sailing with a comprehensive reference from the perspectives of energy.

## 1 Introduction

Due to the extremely vast area of the maritime environment, autonomous robotic systems have been heavily demanded to reduce risk to human and increase efficiency. A large number of such demands remain on the marine surface, such as ocean upper layer observation, pollution detection, patrolling, and communication. (Stelzer and Jafarmadar, 2011; Cruz and Alves, 2008). Vastness of the ocean has placed significant challenges on marine surface robots for long-term operation, especially from perspectives of energy.

Classical unmanned surface vessels (USVs) powered by electricity or fossil fuels in general, though have been widely employed for marine exploration, can hardly work for long in both range and time, due to the limit of energy supply. Wave gliders, from another design perspective, can operate for long term propelled by waves, but have low speed. Sailing robots, propelled by the abundant wind over the sea, have the potential to combine both long-term functionality and satisfying speed. They also provide a carbon-free choice on marine surface transportation. There have been a large number of teams devoting continuously on sailing robots, but only a few of them have successfully completed long missions.

There have been a number of reviews on sailing robots. Stelzer (Stelzer and Jafarmadar, 2011), one of the pioneering researchers in this field, summarized the effort from major groups in 2011. Silva et al. 2019 provided a review 2 years ago but only on rigid-wing sailing robots. This article focuses on the important perspectives of energy for long-term sailing. We group and elaborate the R&D work, main specifications, advantages, and shortcomings comprehensively of the sailing robots developed by each team so as to view their research paths and inspire researchers for deep insights. We analyze from three key energy perspectives, i.e., actuation, electricity harvesting, and energy management. It is hoped that this article can help researchers to obtain the clues or solutions to achieve the objective of long-term robotic sailing.

The rest of this article is organized as follows: Section 2 presents basics and the overview of sailing robots in different groups with detailed configurations. Section 3 elaborates the autonomous sailboats developed from academia, i.e., universities and research institutes. Section 4 presents the work in commercial companies. Section 5 shows the effort from competitions and open communities, which have also boosted R&D from universities, institutes, and companies. Section 6 summarizes and presents some insights for researchers to consider in designing such robots and manage the energy. Section 7 concludes the whole survey.

## 2 An Overview of Sailing Robots

### 2.1 Basics of Sailing Robots

Sailing robots take wind as the main power source with the aim of low-energy consumption. We briefly introduce the sailing robots from four aspects: mechanical structure, sail force analysis, general control scheme, and sailing robot architecture.

**FIGURE 1:**
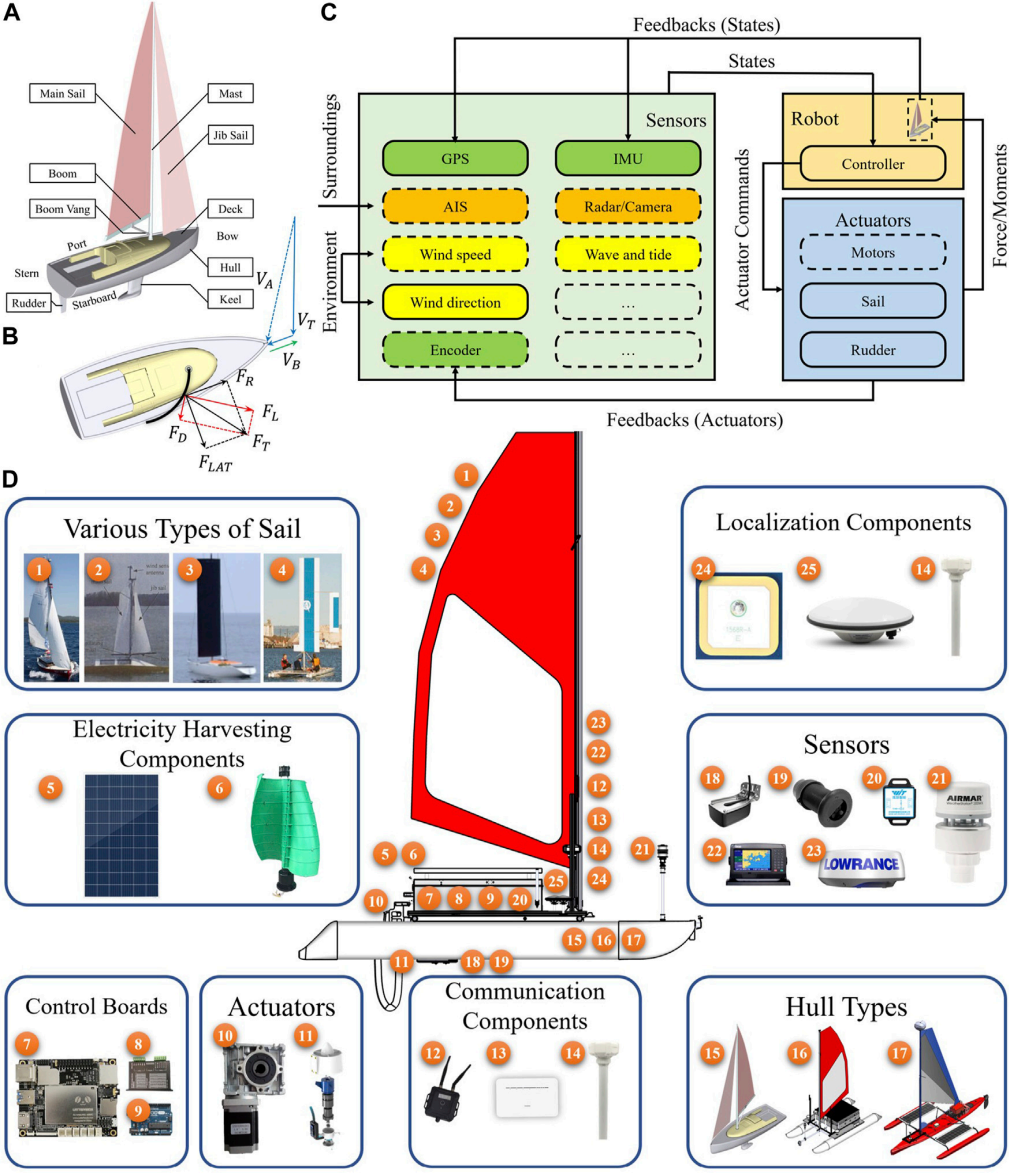
Basic system structure of sailing robots. **(A)** Mechanical structure. **(B)** Sail force analysis. **(C)** General control scheme. **(D)** Typical architecture. In various types of the sail, 1–4 are soft sail, soft sail with a balanced rig, wing-sail, and wing-sail with self-trimming, respectively. For electricity harvesting, the sailing robots are equipped with a solar panel and wind generator in 5, 6. In control boards, there are main control board, motor driver board, and micro-control board for I/O signals in 7–9. Actuators include stepper motors for the sail and rudder and propeller motors in 10, 11. The communication methods consist of wireless radio station, 4G cellular module, and satellite communication station in 12–14. Based on the hull types, the sailing robot can be divided into monohull, catamaran, and trimaran sailboats in 15–17. Classical sensors include underwater sonar, wave current sensor, inertial measurement unit (IMU), weather station, automatic identification system (AIS), and radar with labels 18–23, respectively. The localization systems include Global Positioning System (GPS), Real-time kinematic GPS (RTK-GPS), and radio determination satellite system (RDSS) in 24, 25, and 14.

In Figure 1A, a sailing robot is mainly composed of a sail system, a hull, a rudder, and a keel (optional) (Sailboat, 2021). The sail system, adjusted according to the wind, propels the sailboat forward. It generally includes main sail, jib sail, mast, boom, and boom vang. The hull, a carrier, is described as the bow and stern in different parts. Looking from the stern to the bow, the left side is named port, and the right side is named starboard. The deck is the top ceiling of the hull. The rudder is used to steer the robot. The keel is the load-bearing structure, which not only prevents the robot from lateral drift but also helps maintain its stability.

For sailing robots to locomote in the wind, sailing upwind is the most challenging. In Figure 1B, the wind force acting on the sail during an upwind locomotion is analyzed (SailonForce, 2021). The propulsion of a sailboat depends on the boat speed, heading angle, wind speed, and wind direction. Apparent wind *V*_*A*_ is the speed measured by on-board sensors. It is the vector obtained by true wind speed *V*_*T*_ and the sailboat speed *V*_*B*_. The total force produced by *V*_*A*_ on the sail is *F*_*T*_, which is composed by lift force *F*_*L*_ and drag force *F*_*D*_. Hereby, the total aerodynamic force *F*_*T*_ can be decomposed into the driving force *F*_*R*_ that keeps the sailboat forward and the lateral force *F*_*LAT*_ that pushes the sailboat sideways. The keel can balance the lateral force. Therefore, the sailboat moves forward with the wind propulsion.

In Figure 1C, a general control scheme of the sailing robot is depicted. It is divided into sensors (light green), robot (light gold), and actuators (light blue). The optional components are represented with dashed boxes. The feedback sensors such as GPS, IMU, and encoder are shown in green boxes. The surrounding sensors such as automatic identification system (AIS), radar, and camera are shown in orange boxes. The environmental sensors, for e.g., wind speed, wind direction, and wave and tide sensors are shown in yellow boxes. All states are passed to the controller in the robot. Hereby, the generated force or moment from the controlled actuators will act on the sail, rudder, and propellers.

In Figure 1D, the sailing robot architecture is shown. The typical architecture is decomposed into various types of sail, electricity-harvesting components, control boards, actuators, communication components, hull types, sensors, and localization components. The detail components are represented by corresponding indices. In some recent cases, stepper motors are used to control rudder and sail with worm gearboxes. Propeller motors work as auxiliaries in emergency cases. For different missions, different components in each part can be chosen.

### 2.2 The Overview of Sailing Robots

We summarized the data of existing sailing robots from academia and industry as shown in Tables. 1–4, so that readers can have an overall view.

**TABLE 1:** The sailboats configuration about team information, hull configuration and environment in different research groups. Noted: The column of “Beam/Length” represents the ratio between beam and length; In “Weight (Kg)” column, If the data ended with “(D)”, which is described the data is displacement. Otherwise, the data is the weight of sailing robot.

Team information				Hull Configuration								Environment and performance		
Country	Year	Name	Team	Hull	Length(m)	Beam(m)	Beam/Length	Height(m)	Draft(m)	Weight (Kg)	Payload (Kg)	Wave and wind	Speed (Knots)	Endurance
US	2001	Atlantis	Stanford University &UCSC	Catamaran	7.2	3	-	-	-	-	-	12 ∼20 Knots (Wind)	4	-
UK	2004	AROO	Aberystwyth University	Mono	1.5	-	-	-	-	-	-	-	-	36 H
UK	2006	ARC	Aberystwyth University	Mono	1.5	-	-	-	-	-	-	-	-	-
Austria	2006	ASV Roboat I	INNOC	Mono	1.38	0.34	0.25	1.73	0.24	17.5(D)	-	-	-	-
Austria	2006	ASV Roboat	INNOC	Mono	3.75	-	-	-	-	300	50		4.5	7 H/28 Km
US	2007	HWT-X1	UCSC	Catamaran	9.1		-	10.7	-	-	-	-	-	-
UK	2007	Beagle-B	Aberystwyth University	Mono	3.5	-	-	-	-	-	-	-	-	19 H/25 Km
UK	2008	Pinta	Aberystwyth University	Mono	2.95	1.2	-	-	-	40	-	-	-	18 Days/653 Km
UK	2008	MOOP	Aberystwyth University	Mono	0.74	-	-	-	0.125	4	-	<10 cm (Wave)	0.51	64.65 Hrs
												3.5 ∼16.5 Knots (Wind)		61.27 Km
France	2008	IBOAT	Universite de Toulouse, ISAE	Mono	2.4	0.4	0.17	3	-	35	-	-	3	-
Portugal	2008	FASt	University of Porto	Mono	2.5	0.67	0.27	3.4	1.25	70	-	-	-	-
US	2008	WASP	Florida Atlantic University	Mono	4.2	0.8	0.19	-	1	275(D)	200	-	-	-
US	2008	First Time	United States Naval Academy	Mono	2	0.36	0.18	-	1.5	26.7(D)	-	0 ∼15 Knots (Wind)	-	-
Switzerland	2009	Avalon	ETH	Mono	3.95	-	-	-	2	>160	-	0 ∼30 Knots (Wind)	-	-
France	2009	Breizh spirit	ENSTA	Mono	1.3	-	-	-	0.8	13(D)	-	10 ∼30 Knots (Wind)	3 (Avg)	6 Months
												5.6 (Max)		
US	2009	Luce Canon	United States Naval Academy	Mono	2	0.28	0.14	-	1.5	24(D)	-	0 ∼15 Knots (Wind)	-	-
US	2010	Gill the Boat	United States Naval Academy	Mono	2	0.305	0.15	-	1.5	29.9(D)	-	0 ∼30 Knots (Wind)	-	-
France	2011	Vaimos	ENSTA&IFREMER	Mono	3.65	0.86	0.24	-	0.65	-	90	12 Knots (Wind)	2 ∼5	19 H
														105 Km
France	2011	L’improbable	ENSTA	Mono	-	-	-	-	-	-	-	-	-	-
Germany	2011	rrMM	University of Lübeck	Mono	0.53	0.18	0.34	0.98	-	1.03	-	2 ∼20 Knots (Wind)	2	-
Germany	2011	FHsailbot	FH Stralsund	Mono	1.52	0.33	0.22	-	0.81	15(D)	-	-	-	-
Germany	2011	Saudade	FH Stralsund	Mono	1.12	0.26	0.23	-	0.26	9(D)	-	-	-	-
US	2012	SOA	United States Naval Academy	Mono	2	0.33	0.17	-	1.5	52.2(D)	-	-	-	-
US	2012	W2H	United States Naval Academy	Mono	2	0.48	0.24	-	1.5	44(D)	-	-	2.4	-
Canada	2012	Thunderbird	University of British Columbia	Mono	2	-	-	-	-	-	-	-	-	-
France	2013	MARIUS	Maison des Techologies	Mono	<2	-	-	-	0.8	<100	70	-		-
Spain	2013	ATIRMA	IUSIANI	Mono	1	0.245	0.25	-	0.14	4.3(D)	-	-	-	-
UK/US	2014	ARRTOO	United States Naval Academy	Mono	4.85	-	-	-	0.83	29.5(D)	43	-	4 (Sail)	
			Aberystwyth University										10 (Motor)	
US/UK	2015	MaxiMOOP	United States Naval Academy	Mono	1.2	0.35	0.29	-	0.41	16 ∼23(D)	7	-	-	-
			Aberystwyth University											
Finland	2015	mini12	Aland Univeristy of applied science	Mono	4	-	-	-	-	-	-	-	-	-
Switzerland	2015	AEOLUS	ETH	Mono	-	-	-	-	-	-	-	-	-	-
Spain	2015	ATIRMA G2	IUSIANI	Mono	2	0.37	0.19	-	-	43	-	-	-	-
China	2015	SJTU Sailboat	Shanghai Jiao Tong University	Mono	1.5	0.476	0.32	-	0.433	-	-	0 ∼7.8 Knots (Wind)		-
US	2015	SailVane	Cornell University	Mono	-	-	-	-	-	-	-	-	-	-
France	2015	ASAROME	UPMC	Mono	3.6	-	-	-	-	-	-	5 ∼20 Knots (Wind)	2.5 (Avg)	2 Days
France	2015	ASAROME	UPMC	Mono	3.6	-	-	-	-	-	-	5 ∼20 Knots (Wind)	2.5 (Avg)	2 Days
Portugal	2015	Zarco	University of Porto	Trimaran	2.5	-	-	-	-	50	-	-	-	-
Canada	2016	Ada	University of British Columbia	Mono	5.5	-	-	-	-	-	-	-	-	700 Km
Canada	2016	Raye	University of British Columbia	Mono	-	-	-	-	-	-	-	-	-	-
Finland	2016	mini12	Aland Univeristy of applied science	Mono	4	-	-	-	-	-	-	-	-	-
China	2016	Sail-Based ASV	Smart China Research institute,	Trimaran	5.02	2.9	0.58	-	-	-	-	5.40 Knots (Wind)	3.50 (Avg)	
			Hong Kong&CUHK-Shenzhen										4.67 (Max)	
Finland	2017	ASPire	Aland University of applied science	Mono	4	-	-	-	-	370	-	-	-	-
Sweden	2018	Maribot Vane	KTH	Mono	4.16	0.8	0.19	-	1	≥250	-	3.89 ∼11.66 Knots (Wind)	-	-
UK	2018	Black Python	University of Southampton	Mono	1	0.165	0.17	-	0.42	4(D)	-	-	-	-
China	2018	Hybrid Sailboat-II	CUHK-Shenzhen	Catamaran	0.4	-	-	-	0.6	-	-	2.3 ∼2.7 Knots (Wind)	-	-
Italy	2019	UNIFI	University of Florence	Mono	<2	-	-	-	-	-	<20	-	-	4 Days
China	2020	OceanVoy	CUHK-Shenzhen	Catamaran	3.1	1.4	0.45	-	0.2	75	200	Sea state 3 (Wave)	3 (Avg)	>7 Days
												2 ∼25 Knots (Wind)	4.98 (Max)	

**TABLE 2:** The sailboats configuration about propulsion source, energy harvesting and energy management in different research groups.

	Propulsion source						Energy harvesting			Energy management		
Name	Sail Type	Special Sail Design	Sail (mˆ2)	Sail (m*m)	Motor	Wave Glider	Solar(W)	Wind(W)	Wave(W)	Battery (Wh)	Total Power Consumption(W)	Method
Atlantis	Wing Sail	Self-trimming	17	-	Yes	-	-	-	-	-	-	-
AROO	Wing Sail	-	-	-*1	-	-	-	-	-	50.4	10	Artificial Endocrine Controller
ARC	Wing Sail	Double Sails	0.02	0.07*0.3	-	-	-	-	-	60	10	Artificial Endocrine Controller
ASV Roboat I	Soft Sail	Four Sails	0.855	-	-	-	-	-	-	-	-	-
ASV Roboat	Soft Sail	Balanced Rig	4.5	-	-	-	285	-	-	1920	35	-
HWT-X1	Wing Sail	Self-trimming	-	-	-	-	-	-	-	-	-	-
Beagle-B	Wing Sail	-	3.5	-*4	-	-	30	-	-	2,880	1.777 7	Artificial Endocrine Controller
Pinta	Soft Sail	-	5.39	-	-	-	120	-	-	1,344	9.175	Artificial Endocrine Controller
MOOP	Wing Sail	-	0.02	0.07*0.3	-	-	4.75	-	-	55	10	Artificial Endocrine Controller
IBOAT	Soft sail	Balanced rig	1.5	-	-	-	20 (1st)	-	-	-	<7 (Avg)	-
							90 (2nd)					
FASt	Soft Sail	-	3.7	-*3.4	-	-	45	-	-	190	1.85	-
WASP	Wing Sail	-	-	-	Yes	-	25	-	-	2000	105	-
First Time	Soft Sail	-	3.1	-	-	-	-	-	-	>13.2	-	-
Avalon	Soft Sail	Balanced Rig	8.4	-	-	-	360	-	-	600	40	-
Breizh Spirit	Soft Sail	-	0.86	-	-	-	-	-	-	144	-	-
Luce Canon	Soft Sail	-	3.1	-	-	-	-	-	-	>13.2	-	-
Gill the Boat	Soft Sail	-	3.1	-	-	-	1.8	-	-	208.8	-	-
Vaimos	Soft Sail	Balanced Rig	-	-	-	-	-	-	-	-	-	-
L’improbable	Soft sail	Wind vane	-	-	-	-	-	-	-	-	-	-
		self steering										
rrMM	Soft Sail	-	-	-	-	-	-	-	-	-	-	Optimal Sail and Rudder
FHsailbot	Soft Sail	-	0.65	-*2	-	-	-	-	-	-	-	-
Saudade	Soft sail	-	0.52	-	-	-	-	-	-	-	1.8 (Min)	
											20(Peak)	
SOA	Soft Sail	Balanced Rig	1.9	-	-	-	10.8	50	-	1,296	7.2	Sampling Frequency
W2H	Soft Sail	Balanced Rig	1.8	-	-	-	10.8	50	-	1728	7.2	Sampling Frequency
Thunderbird	Soft Sail	-	-	-	-	-	-	-	-	-	-	-
MARIUS	Soft sail	-	2.9	-*2.4	-	-	70	30	-	-	<23	Sampling frequency
												Multi-mode
ATIRMA	Soft Sail	-	0.61	-*1.6	-	-	-	-	-	42.09	1.26 (Avg)	-
ARRTOO	Soft Sail	Double Sails	-	-	Yes	-	260	120	-	-	4.187	-
MaxiMOOP	Soft Sail	-	0.24 ∼1	-	-	-	-	-	-	-	-	
mini12	Soft Sail	-	-	-	-	-	-	-	-	-	-	Multi-Mode
AEOLUS	Soft Sail	-	-	-	-	-	-	-	-	-	-	-
ATIRMA G2	Wing Sail	Double Sails	0.64	-	-	-	-	-	-	-	-	-
SJTU Sailboat	Soft Sail	-	1.15	-	-	-	-	-	-	-	-	3DDP
SailVane	Wing sail	Self-trimming	-	-*1.3	-	-	29.57	-	-	-	4.48 (Max)	Two-mode
											0.18 (Min)	
ASAROME	Soft Sail	-	-	-*2.25	-	-	60	50	-	1,440	35 (Avg)	Rudder PD controller
Zarco	Wing Sail	-	0.3	0.3*1	Yes	-	-	-	-	-	-	-
Ada	Soft Sail	-	-	-	-	-	-	-	-	-	-	-
Raye	Soft Sail	-	-	-	-	-	-	-	-	-	-	-
mini12	Wing Sail	Self-trimming	8.2	-	-	-	-	-	-	-	-	-
Sail-Based ASV	Soft Sail	-	5.47	1.99*4.64	Yes	-	440	-	-	2,400	-	-
ASPire	Wing Sail	Self-trimming	-	-	-	-	50	-	-	1,320	-	-
Maribot Vane	Wing Sail	Self-trimming	-	-	Yes	-	-	-	-	-	-	-
Black Python	Soft Sail	-	≤0.6	-	-	-	-	-	-	-	-	-
Hybrid Sailboat-II	Soft Sail	-	0.079	-*0.5	Yes	-	-	-	-	5.55	<7	-
UNIFI	Soft Sail	-	-	-	-	-	99	-	-	480	4	-
OceanVoy	Soft Sail	-	3.75	-	-	-	180	-	-	5,760	30	E-saving method

**TABLE 3:** The sailboats configuration in different companies (Part 1).

Team information				Hull Configuration								Environment and performance		
Country	Year	Name	Team	Hull	Length(m)	Beam(m)	Beam/Length	Height(m)	Draft(m)	Weight (Kg)	Payload (Kg)	Wave/Wind	Speed (Knots)	Endurance
Norway	2018	Sailbuoy	Offshore sensing AS	Mono	2	-	-	1.13	0.57	60	10	>15 m (Wave)	1 ∼3	Serveral months
												5.8 ∼58.3 Knots (Wind)		
Norway	2018	Sailbuoy Wave	Offshore sensing AS	Mono	2	-	-	1.13	0.57	55	10	>8 m (Wave)		Serveral months
												5.8 ∼58.3Knots (Wind)		
US		Saildrone Explorer	Saildrone	Mono	7	-	-	-	2	-	-	-	3	12 Months
US		Saildrone Voyager	Saildrone	Mono	10	-	-	-	2	-	-	-	5	3 Months
US		Saildrone surveyor	Saildrone	Mono	22	-	-	-	3	-	-	-	6	>6 Months (Under sail)
														4630 Km/6 Knots (Under power)
Australia		BlueBottle	OCIUS	Mono	6.8	-	-	-	-	-	600	<sea state 6	5	-
US	2019	Datamaran Mark 7	Autonomous Marine System	Catamaran	3.7	-	-	-	0.3	192	23	5.8 ∼40.8 Knots (Wind)	-	6 Months
US	2019	Datamaran Mark 8	Autonomous Marine System	Catamaran	5	-	-	-	0.8	360	70	5.8 ∼40.8 Knots (Wind)	-	6 Months
US		Submaran	Ocean Aero	Mono	4.4	0.8	0.18	3	1.5	350	-	-	5 (Surface)	>3 Months (Surface)
													2 (Subsurface)	>8 Days (Subsurface)
Japan		Type A	EverBlue	Trimaran	2	-	-	-	-	-	-	1.4 ∼14.62 Knots (Max)	0.78 (Avg)	
													3.65 (Max)	
US		Gen6/7	SubSeaSail	Mono	1.52	0.25	0.16	3.04	1.3	28	20	3 ∼5 m (Wave)		
												<30 Knots (Wind)		

**TABLE 4:** The sailboats configuration in different companies (Part 2).

	Propulsion source						Energy harvesting			Energy management		
Name	Sail Type	Special Sail Design	Sail (mˆ2)	Sail (m*m)	Motor	Wave Glider	Solar (W)	Wind (W)	Wave (W)	Battery (Wh)	Total Power Consumption (W)	Method
Sailbuoy	Wing Sail	-	0.4/0.6	0.52*-	-	-	40	-	-	400	-	Only rudder actuated
Sailbuoy Wave	Wing Sail	-	0.4/0.6	0.52*-	-	-	30	-	-	400	-	Only rudder actuated
Saildrone Explorer	Wing Sail	Self-trimming	-	-*5	-	-	Yes	-	-	-	-	-
Saildrone voyager	Wing sail	Self-trimming	-	-*6	4 Kw	-	Yes				300 (Avg)	
											2000 (Peak)	
Saildrone surveyor	Wing sail	Self-trimming	-	-*13	56.25 Kw	-	Yes	-	-	-	2000 (Avg)	-
											3,000 (Peak)	
BlueBottle	Wing Sail	Foldable	-	-	Yes	Yes	Yes	-	-	-	50	Fold Sail (>sea state 6)
Datamaran Mark 7	Wing sail	Foldable	6.2	2*3.1	-	-	Yes	-	-	-	20 (Avg)	-
											1,000 (Peak)	
Datamaran Mark 8	Wing sail	Foldable	13.2	3.3*4	Yes	-	Yes	-	-	-	95 (Avg)	-
											200 (Peak)	
Submaran	Wing Sail	Foldable	-	-*3	-	-	200	-	-	4,000	-	-
Type A	Soft Sail	-	-	-	-	-	-	-	-	-	-	-
Gen6/7	Wing sail	Passived	-	-	Yes	-	30(Wingsail)	-	-	450	5 (Avg)	Self-regulating Wingsail
							10 (Deck)				20(Peak)	

**FIGURE 2:**
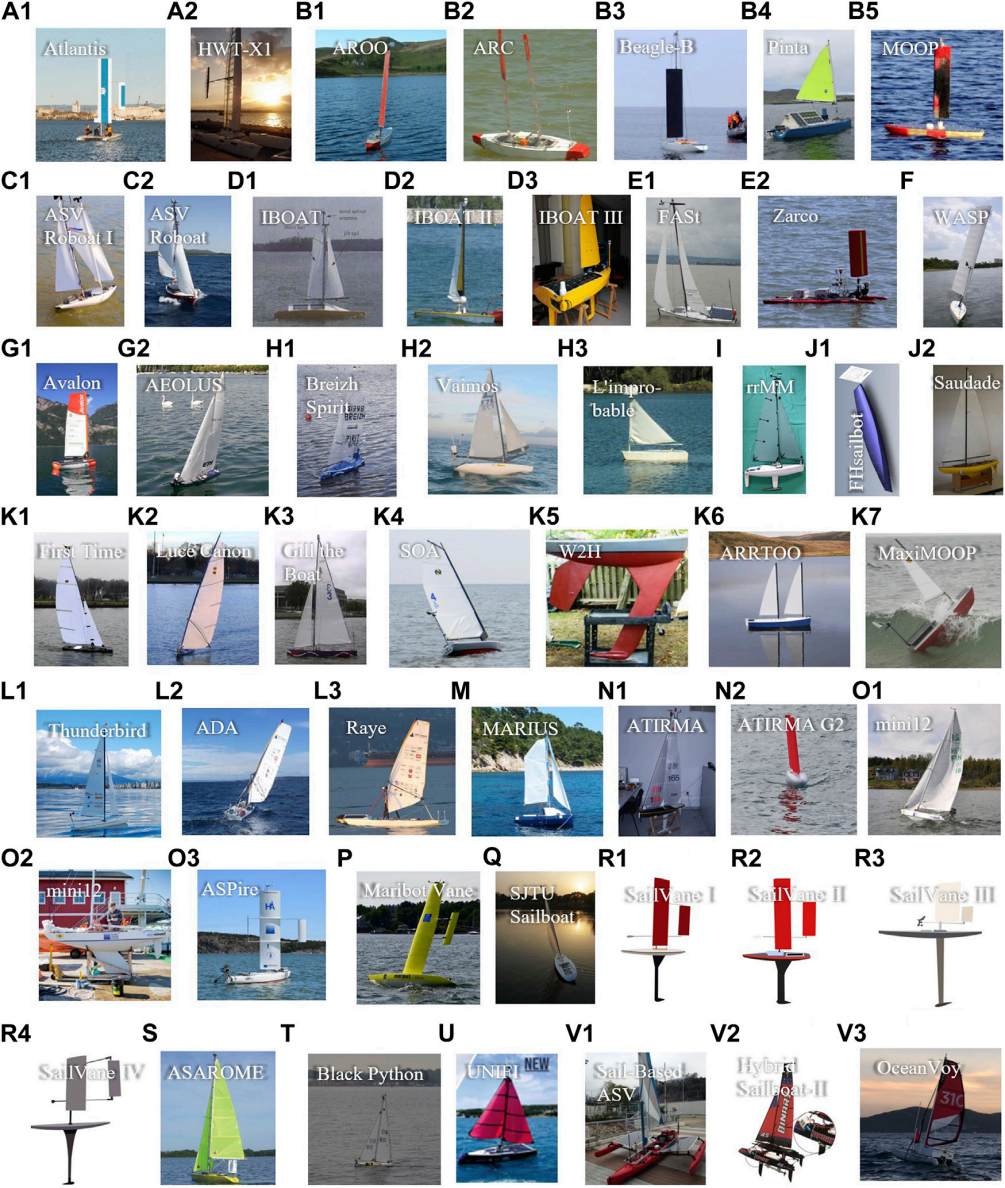
Sailing robots from universities and research institutes. **(A)** Stanford University and University of California, Santa Cruz (UCSC), USA. **(B)** Aberystwyth University, UK. **(C)** Austrian Society for Innovative Computer Sciences (INNOC), Austria. **(D)** Universite de Toulouse, ISAE, France. **(E)** University of Porto, Portugal. **(F)** Florida Atlantic University (FAU), US. **(G)** ETH, Switzerland. **(H)** École Nationale Supérieure de Techniques Avancées (ENSTA), France. **(I)** University of Lübeck, Germany. **(J)** FH Stralsund, Germany. **(K)** United States Naval Academy (USNA), USA. **(L)** UBC Sailbot, Canada. **(M)** Institut Supérieur de Mécanique (SUPMECA) and Institut Supérieur de l’Electronique et du Numérique (ISEN), France. **(N)** Instituto Universitario SIANI (IUSIANI), Spain. **(O)** Åland University of Applied Sciences, Finland. **(P)** KTH Royal Institute of Technology (KTH), Sweden. **(Q)** Shanghai Jiao Tong University (SJTU), China. **(R)** Cornell University, US. **(S)** Univ Paris 06 (UPMC), France. **(T)** University of Southampton, UK. **(U)** University of Florence, Italy. **(V)** The Chinese University of Hong Kong, Shenzhen, and Shenzhen Institute of Artificial Intelligence and Robotics for Society (AIRS), China.

In Figure 2, numerous research teams from academia have contributed continuously in robotic sailing, and 22 teams are collected in this review. They have proposed new designs for actuation, tried different methods to harvest energy, and explored a number of strategies to manage energy for higher efficiency. The sailboats developed by each team are grouped by a letter.

**FIGURE 3:**
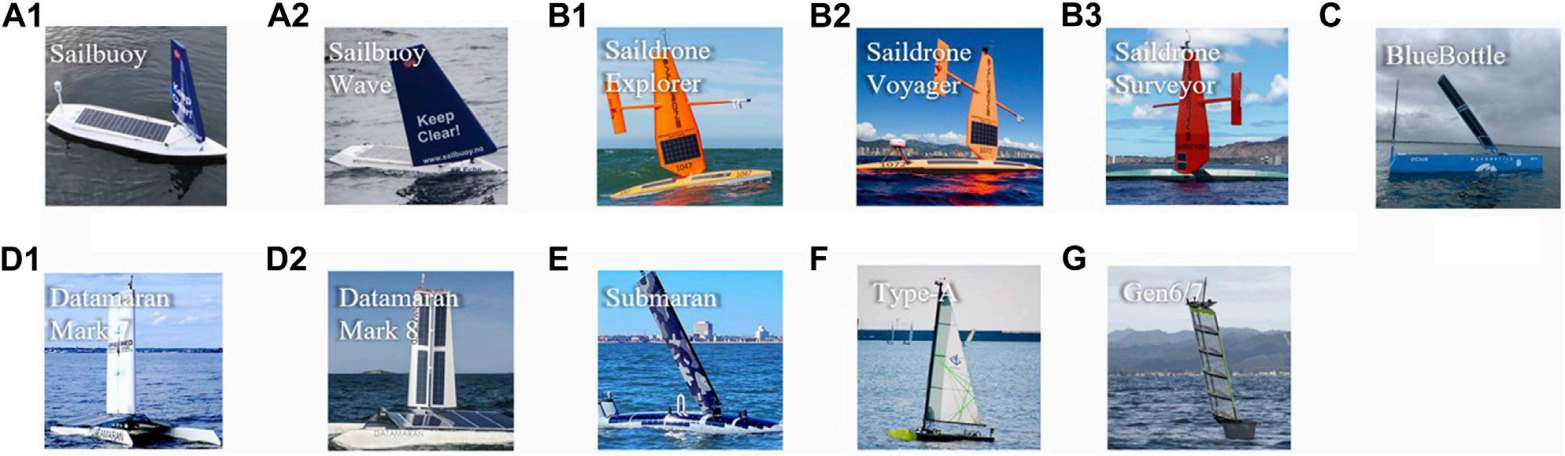
Sailboats in existing sailboat groups from commercial companies. **(A)** Offshore Sensing AS, Norway. **(B)** Saildrone, Inc., USA. **(C)** OCIUS, Australia. **(D)** Autonomous Marine System (AMS), USA. **(E)** Ocean Aero, USA. **(F)** Everblue, Japan. **(G)** SubSeaSail, USA.

In Figure 3, visible achievements from industry are illustrated. Offshore Sensing AS and Saildrone Inc. have deployed robust sailing robots in the ocean and accumulated considerably long-distance voyage.

**FIGURE 4:**
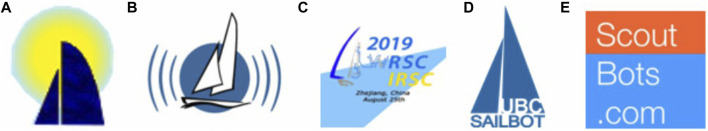
Different sailboat groups in society and organization. **(A)** Microtransat Challenge, 2006–present. **(B)** International Robotic Sailing Regatta (IRSR) SailBot, 2006–present. **(C)** World Robotic Sailing Championship (WRSC) and International Robotic Sailing Conference (IRSC), 2008–present (The logo is chosen from the 2019 activity). **(D)** Scoutbot community.

In Figure 4, a few years after debut of sailing robot prototypes, ambitious competitions were organized, and some became excellent platforms encouraging researchers for long-term sailing, and some boosted technologies in design, intelligence, etc. An open technology community has also shared knowledge and attracted global researchers into robotic sailing.

**FIGURE 5:**
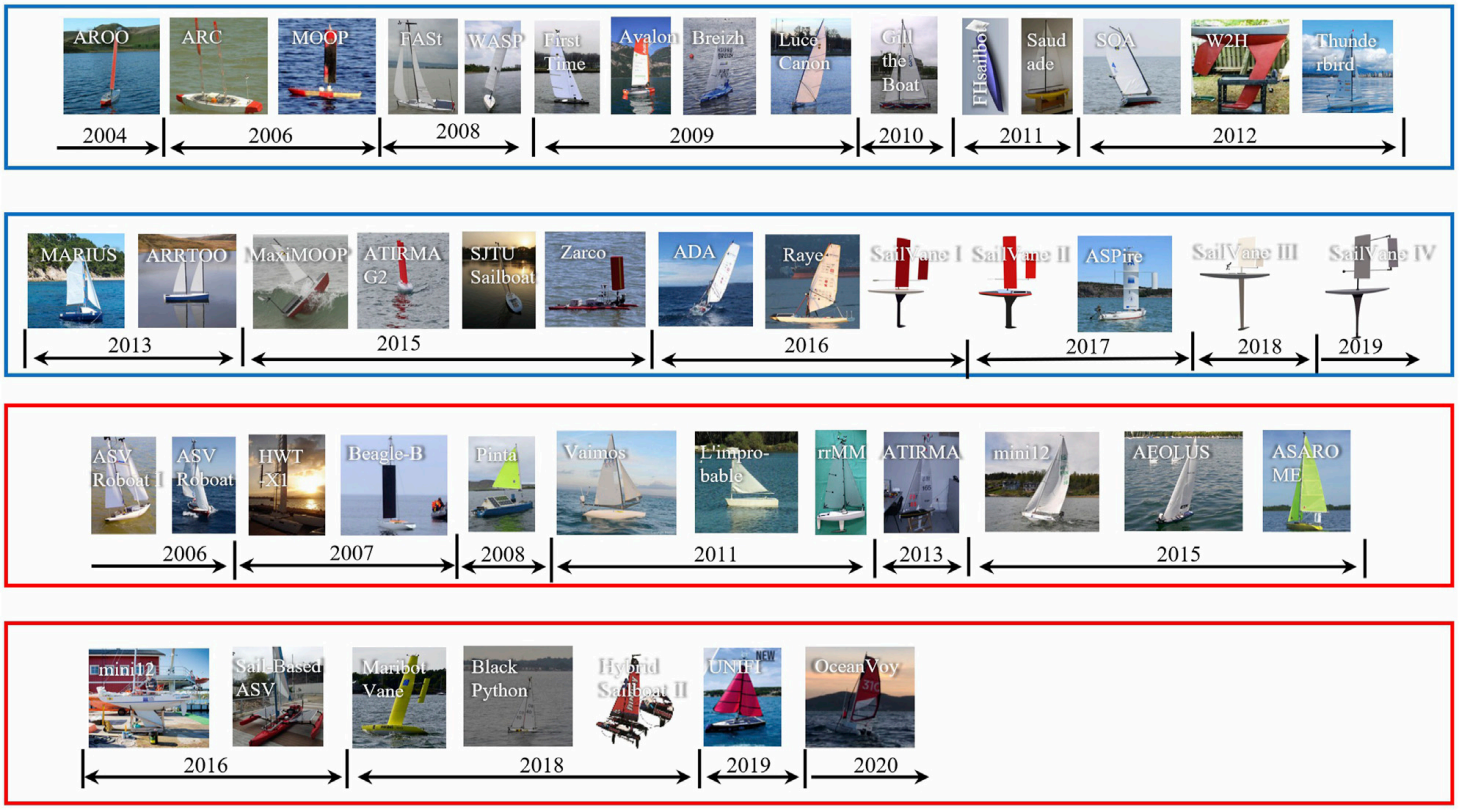
Two groups of developed sailboats: designing by researchers (in blue boxes) and retrofitting based on retailing sailboats (in red boxes).

There are two types of ideas to develop the main bodies of sailing robots as shown in Figure 5. One way is for researchers to start from the sketch and design hull, keel, and sails all by their own, as shown in the blue boxes. Another approach is to retrofit based on off-the-shelf sailboats, as marked in the red boxes. In recent years, the retrofitting methodology have become very attractive, mainly due to the shortened development cycle and reduced cost.

Moreover, sailboats can be divided into two groups, i.e., soft sail and wing sail. In academia, more sailing robots, i.e., 33 have adopted soft sails, while 13 choose wing sails. Wing sails, however, are more attractive to commercial companies.

## 3 Sailing Robots From Academia

Various research teams have been devoted to robotic sailing. We elaborate the effort of each team chronologically for researchers to clearly view their progression.

### 3.1 Stanford University and University of California, Santa Cruz, USA

The Atlantis in Figure 2 (A-1) (Elkaim 2002; Elkaim 2006; Elkaim 2009) was an autonomous catamaran sailboat initially built in Stanford University and later further explored in the UCSC as Elkaim graduated and moved there. It was based on a Prindle-19 catamaran with 7.2 m long, 3 m wide, and was equipped with a 17-m^2^ wing sail. The wing sail was designed innovatively with a flying tail to enable self-trimming for an optimal angle automatically.

The Atlantis was designed to demonstrate a very high precision of navigation and control, even in the presence of wind and waves. This robot was tested in Redwood City harbor on January 27, 2001, for closed-loop control with approximately 12knots (or 6 m/s) of wind, and gusted up to the 20knots (or 10 m/s) range. A few tens of path segments were recorded and compared with the paths by a human sailor. One challenge on the Atlantis was that multiple humans were required as ballast to prevent it from capsizing.

After the successful design and implementation of the Atlantis project, the researchers extended the navigation algorithms and demonstrated a more complete architecture for vehicle control. The HWT X-1 in Figure 2 (A-2) (Boyce and Elkaim, 2007; Elkaim and Boyce, 2007) was a 9.1-m (30 ft) catamaran, with a carbon fiber wing of 10.7 m^2^. Two flying tails were mounted on the boom, so as to obtain the optimal angle. Electric motors were also included to propel the sailboat under an insufficient wind environment. Short distance experiments in both the protected water and open sea in Hawaii were conducted to validate the effect of line tracking.

Although it is unknown if the authors have further improved the sailing robots for long-distance/time sailing, for e.g., in the aspect of energy harvesting, energy management, etc., these efforts initialized the inspiring design of wing sail for precise motion control.

### 3.2 Aberystwyth University, UK

Aberystwyth University has designed and developed a series of sailing robots since 2004, including Autonomous Robot for Ocean Observation (AROO), Autonomous Robotic sailing Craft (ARC), Beagle-B, Pinta, and Miniature Ocean Observation Platform (MOOP) in Figure 2 (B-1–B-5).

They started with the AROO, which was a monohull 1.5-m long sailboat with one aluminum wing sail, aiming to prevent the break or jam of rope required to control traditional fabric sail. Two tests were conducted in a small lake, and issues, for e.g., frequent overshooting in sail control and long rudder action time were raised (Sauze and Neal, 2006). In 2006, ARC was developed, with a monohull in a similar dimension, but two lighter wing sails were made from acrylic and wood (Neal et al., 2009). In the test, when the two sails were positioned appropriately, ARC demonstrated stable-sailing capability and followed a straight course, even if the control system was off. This showed the potential to further reduce power consumption (Sauze and Neal, 2006). In (Benatar et al., 2009), Neal collaborated with researchers from the University of Nottingham, University of York, and University of Reading and designed a P-controller as an expert system for maneuvering rudderless sailboats with two masts that can steer with sails only.

To enhance the capability for long-period sailing, a 3.5-m long sailing dinghy was retrofitted into Beagle-B (Sauze and Neal, 2008). Dual wing sails could not fit the dingy hull layout, and thus single wing sail was adopted. Two 15 W solar panels were placed on the deck to charge batteries. An average power consumption at 1.777 7 W was estimated. A total distance of 25 km over 19 h was traveled in the Microtransat Challenge 2007.

Targeting to cross the Atlantic Ocean and prevent from losing the expensive Beagle-B, another sailboat Pinta was built based on the Topper Taz child’s sailing dinghy. Fabric sail replaced wing sail to ease construction and reduce cost. 120 W peak photo-voltaic solar panels were fixed on the sloped surface. An average total power at 9.175 W was estimated (Sauze and Neal, 2010). Pinta, although finally lost in the Microtransat Challenge 2010, was remarkably the first sailing robot attempting transatlantic and reached 87 km autonomously in the 18-day journey (Microtransat, 2010).

The MOOP (Sauzé and Neal, 2011), with its R&D work starting from 2008, was a type of 0.72-m long sailboat integrating the advantages in both AROO and ARC. Its small, cheap, robust, and light-weighted features were attractive to the team, motivated by building a fleet of MOOPs and increasing the probability of crossing the Atlantic, though had not participated yet. One single wing sail and a 4.75 W solar panel were adopted (Sauze and Neal, 2013).

**FIGURE 6:**
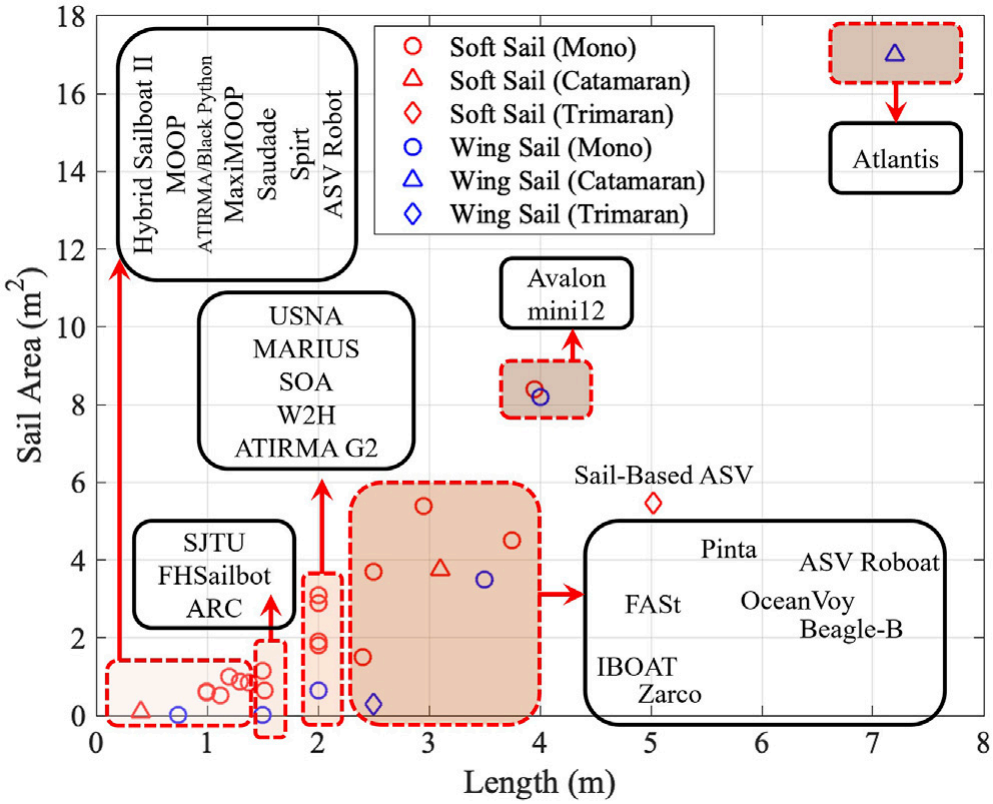
Correlation between sail area and sailboat length.

**FIGURE 7:**
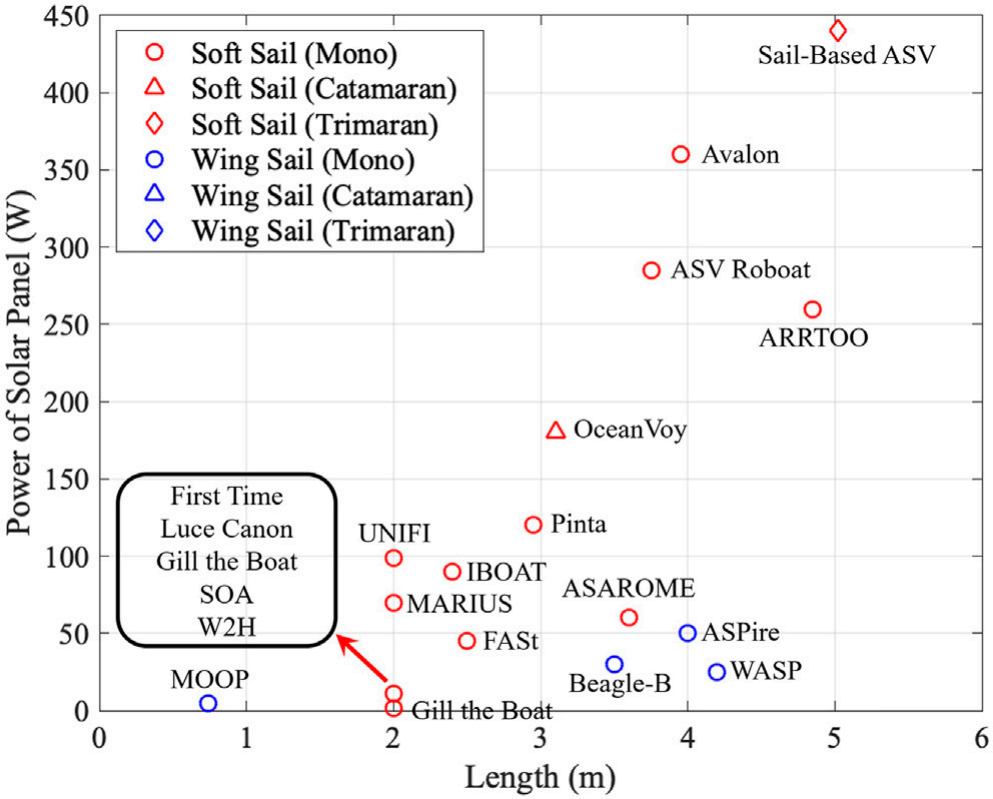
Correlation between sailboat length and Watts on solar panels.

The Aberystwyth University group started to collaborate with the United States Naval Academy (USNA) team in building the Autonomous Robot for Rapid Transit and Ocean Observation (ARRTOO) shown in Figure 2 (K-6) (Miller et al., 2014). After that, they also worked together with MaxiMOOP shown in Figure 2 (K-7) (Miller et al., 2015) and upgraded it for the SailBot competition. The details can be found in Section 3.11.

Based on the iterative version of the MOOP, the researcher developed a biologically inspired control and power management method called artificial endocrine controller (Sauze and Neal, 2010; Sauze and Neal, 2011; Sauze and Neal, 2013). It showed potential as a method for energy management demands, gradually switching between behaviors, synchronizing behavior with external events, and maintaining a stable internal state of the robot. Their work showed that applying endocrine-inspired modulation to a neural network offered a powerful mechanism for controlling power consumption in robotic systems.

### 3.3 Austrian Society for Innovative Computer Sciences, Austria

Initially, the team had joint efforts from both Austria and UK, when Stelzer conducted PhD thesis research in De Montfort University, UK. The first autonomous sailboat, named ASV Roboat I in Figure 2 (C-1), was developed from a commercial and remotely controlled model sailboat Robbe Atlantis (Stelzer and Pröll, 2008). It was 1.38 m long and 1.73 m tall, equipped with 2 masts and 4 sails achieving 0.855 m^2^ area to capture wind. They improved the short course routing method from a classical boat speed polar diagram into a binary simple polar diagram–based method and demonstrated its advantage in reducing time by reaching targets in both simulations and experiments. Then, the team extended the research results to a larger ASV Roboat in Figure 2 (C-2). It was retrofitted from the commercially available boat type Laerling (Cruz and Alves, 2010), with the length of 3.75 m and a 60-kg keel-ballast which kept the boat upright. It featured a conventional sloop rig, with 4.5 m^2^ as the total sail area. They developed a balanced rudder (Stelzer and Jafarmadar, 2012) to improve its efficiency and studied the balanced rig (Stelzer and Dalmau, 2013) to reduce the energy consumption on controlling the jib sail. The effect of a balanced rig on the power consumption of a robotic sailboat was investigated, and approximately 68*%* power was saved theoretically on the sail drive through simulation. The average power consumption of this robot was approximately 35 W, while solar panels provided a 285 W energy source at peak and about 30 W in average. The ASV Roboat completed a distance of 28 km for about 7 h in the endurance race at WRSC2010 on Lake Ontario, Canada (Stelzer, 2012).

On long-term routing, Stelzer collaborated with Langbein in Ulm University, Germany, and presented an A* algorithm, considering the changing weather conditions. It was advantageous in short computation time compared with existing commercial approaches (Langbein et al., 2011).

### 3.4 Université de Toulouse, ISAE, France

Briere developed an autonomous sailing robot IBOAT in Figure 2 (D-1) (Briere, 2008) aiming to long-term offshore operation as an enhancement for traditional drifting buoys. It was a monohull sailboat with 2.4 m length. The two sails (main sail and jib sail) were designed in a balanced manner, and only one actuator was needed. To increase robustness in strong wind, a total sail area of 4 m^2^ was reduced to 1.5 m^2^. A maximum power of 90 W solar panels were adopted with an average of 10 W expected to charge the battery. To increase the energy regeneration efficiency, the system was equipped with an MPPT (Maximum Power Point Tracking) function. IBOAT attended the Microtransat Challenge 2006 and 2007.

Thereafter, two other prototypes were developed, namely IBOAT II in Figure 2 (D-2) and IBOAT III in Figure 2 (D-3). The second version further reduced the sail area to 0.8 m^2^ preventing it from instability in strong wind. Solar cells with peak power 80 W and average power 13 W were updated. An average power consumption of 7.68 W was measured, which seemed promising to reach energy balance. IBOAT III, the team’s latest sailboat, was shortened to 1.8 m length and changed to a rigid sail. A 0.65-m^2^ solar panel was equipped. To further evaluate the feasibility to provide energy by the solar panel, sail shadow and boat tilting due to wave, wind, and solar irradiance were considered, and a simulator was built. It is concluded that the average harvested solar energy exceeds the average power consumption by 6 W (Genet et al., 2019).

### 3.5 University of Porto, Portugal

The first autonomous sailing robot developed by the University of Porto, Portugal, was FASt in Figure 2 (E-1) (Alves and Cruz, 2008). It was a 2.5 m long autonomous unmanned sailboat, equipped with a main sail and a jib sail summing up to 3.7 m^2^ total area. The team deployed a 45 W solar panel and a set of Li-ion batteries with 190 Wh total capacity.

Based on FASt, the team proposed a mission programming system for an autonomous sailboat on long-term tasks (Alves and Cruz, 2014). Waypoints setting, events defining, action assigning, and dynamic mission were created in this system. Combining mission planning, supervision, and analysis together, Cruz and Alves later developed an interactive graphic console METASail (Mission Emulation, Tracking and Analysis for Sailing robots) (Alves and Cruz, 2015).

After the FASt project, Cruz integrated a 1 m high rigid wing sail into a 1.5 m long electric ASV named Zarco (Cruz et al., 2015). It was able to utilize wind propulsion in case of low power.

### 3.6 Florida Atlantic University, US

A team at Florida Atlantic University (FAU) developed wind- and solar-powered (WASP) unmanned surface vehicle (ASV) in Figure 2F in 2007–2008. It was a monohull sailboat with a length of 4.2 m, a beam of 0.8 m, a draft of 1 m, and a maximum speed of about 5 knots. It was mainly propelled by wing sails (Baker et al., 2008; Rynne and von Ellenrieder, 2008; Rynne and von Ellenrieder, 2009). One of its main goals was to minimize the power demand of the system toward predicting environmental events and tracking the distribution of meteorological and oceanic conditions over long periods of time. It was equipped with 2,000 Wh energy reservoirs with 24 V battery supply and a 25 W solar panel. In continuous usage, the system consumed over 100 W, in which the power of control/sensors, wing sail motor, and rudder motor were 30 W, 50, and 25 W, respectively.

### 3.7 Swiss Federal Institute of Technology in Zurich (ETH), Switzerland

For the aim to cross the Atlantic Ocean fully autonomously, Siegwart led the team and developed AVALON in Figure 2 (G-1), which was a monohull sailboat robot with length 3.95 m and a single balanced sail of 8.4 m^2^ (Giger et al., 2009; Erckens et al., 2010). A total of two square meters of solar panels were adopted to provide a total 360 W peak power. A direct-methanol fuel cell worked as a backup to charge the battery, when the voltage dropped under a certain value.

Based on the currently available energy and the expected future solar power harvesting, the robot (Frey, 2009) made decisions on how much energy to spend. Hence, it could maximize its minimal service level.

From the start in each day, the power management system processed and operated for every day. Comparing different algorithms, the simple mixed integer linear program (MILP)–based algorithm outperformed others in the weather forecast scenarios. Thus, it was chosen and implemented in the power management system.

Besides, they considered fuel energy as an alternative solution when the system power was not sufficient with a designed energy switch (Siegwart, 2009). AVALON was tested for several shortruns on Swiss lakes and the Atlantic Ocean under the wind 0–30 knots (Erckens et al., 2010).

After AVALON, an autonomous 1-m RC-model sailboat named AEOLUS in Figure 2 (G-2) (Tranzatto et al., 2015) was developed. It did not aim for long time or long range. They focused on designing control architecture rather than energy-harvesting or energy-saving methods. The architecture allowed the robot to sail upwind fast and tack smooth. The test was conducted in Lake Zurich. A high-level controller based on cost function was built, which performed multi-objective optimization of the sailboat trajectory (Wirz et al., 2015).

### 3.8 École Nationale Supérieure de Techniques Avancées, France

The ENSTA team started from the control of a sailboat in theory. Jaulin characterized the polar speed diagram based on interval analysis with collaboration from Herrero in Universitat de Girona, Spain (Herrero et al., 2005), combined a quantified set inversion (QSI) solver with feedback control and verified the method by controlling sailboat speed and orientation in simulation (Herreroa et al., 2008). For WRSC 2009, the team proposed a homemade 1.5 m long sailboat, named Breizh Spirit 1 in Figure 2 (H-1), which had a mono-hull, a main sail, and a jib sail, based on International Monohull Open Class Association (IMOCA) class design. The robot was successfully tested by crossing the Bay of Brest (6.5 nautical miles path) and going upwind around the US island (12 nautical miles path) but finally crashed on rocks because of the inability to tack in strong wind up to 30 knots. To improve from the first prototype, two more sailing robots, Breizh Spirit 2 (2.3 m long) and 3 (1.7 m long) were developed for different purposes in research and Microtransat, respectively (Leloup et al., 2011). The hull design and robust electronics design were updated. As stated from the team, they were able to resist to strong storms, to follow a predefined route, to supply its own energy, and to navigate in sea waves. A 194 km path was covered during the 194 h voyage in Microtransat 2010.

In 2011, to reduce the energy consumption, the ENSTA Bretagne team proposed a solution to self-steer the sailboat in different wind directions and without a wind sensor. Concepts were inspired from wind-vane self-steering system in real sailboats, but they put it in the bow to simplify the design. Simulation validated the innovation, and a sailing robot L’improbable in Figure 2 (H-3) (Sliwka et al., 2011) was developed based on an Optimist type sailboat to test. To increase robustness even more, the sail was tuned to a fixed and empirical angle rather than actuated by a motor. The robot was tested in Ty-Colo Lake.

Collaborating with IFREMER (Institut Francais de Recherche pour l’Exploitation de la Mer), the ENSTA team later developed a 3.65-m sailing robot, named Vaimos in Figure 2 (H-2) (Le Bars and Jaulin, 2013), with the goal of oceanographic measurements. Mini-J hull with a self-righting feature and design of a balanced rig soft sail was adopted. The interval-based method proposed in Jaulin and Le Bars 2012 was integrated into the robot. It traveled 105 km between Brest and Douarnenez in 19 h with approximately 12 knots wind and validated its functionality (Le Bars and Jaulin, 2013). As the researchers focus on the oceanographic measurements and the size of the hull was large enough, the energy-harvesting method such as the solar panel in Vaimos was not considered. A balanced sail indeed reduced energy consumption.

Jaulin further proposed a method to harvest electricity from the regenerative action by the sail motor, when the sail was pushed open by the wind. The generation of electricity functioned only in the downwind path. Based on the parameter of Vaimos, the average collected power can be around 93 W in simulation, while not yet demonstrated experimentally.

### 3.9 University of Lübeck, Germany

Schlaefer, based on a monohull kit (i.e., Graupner Micro Magic Kit), developed small, lightweight sailing robots, named robotic racing Micro Magic (rrMM) (Schlaefer et al., 2011) in Figure 2I, with proven sailing performance. These sailboats were approximately 1.03 kg and with length 0.53 m. The rrMM project did not emphasize on energy problem research. However, it helped groups focus more on the algorithms. In Hertel and Schlaefer 2013, they studied and obtained the optimal sail and rudder via the data-mining method.

\Since 2013, Schroder started the work based on MaxiMOOP with a balanced swing rig in two sails, approximately 0.5 m^2^, and reduced energy consumption (Schröder and Hertel, 2014). It was still far from participating in the Microtransat Challenge, and the energy consumption was analyzed as 10 times more than planned.

### 3.10 FH Stralsund, Germany

FHsailbot in Figure 2 (J-1) is a 1.52-m sailboat (Koch and Petersen, 2011), based on an AMYA (Ammann et al., 2010), 1 m class specification with sail area 0.65 m^2^. However, due to the limitation by rig and transportation problems, they equipped an old model sailboat Saudade in Figure 2 (J-2), with 1.12 m length and 0.52 m^2^ sail area. They adopted ARM7 and *μ*C/OS-II with a minimum total current of about 0.36 A and maximum total current about 4 A at 5 V. Based on the current and voltage data, the working power was about 1.8–20 W.

The FHsailbot was proposed and prepared for WRSC2011 competitions. They did not focus on energy harvesting or management for long-term sailing. However, the low-cost and energy-saving electronics configuration can help the other researchers.

### 3.11 United States Naval Academy, US

The USNA team was formed in January 2007, and they designed a sailboat named *First Time* in Figure 2 (K-1) to participate in the 2008 SailBot race. Their experience helped them to build the second sailboat *Luce Canon* in Figure 2 (K-2) for the 2009 SailBot competition (Miller et al., 2009). In 2010, they designed the third USNA SailBot, named *Gill the Boat* in Figure 2 (K-3), to handle strong wind and waves (Miller et al., 2010). The fourth USNA sailing robot, named *Spirit of Annapolis (SOA)* in Figure 2 (K-4), is also a 2-m sailboat equipped with three 12 V, 36 Ah, and 2.2 kg Shorai batteries (Miller et al., 2013). It was not easy to complete a tack due to high-directional stability. So, they modified the sailboat into the fifth sailing robot called *W2H* in Figure 2 (K-5), in which they upgraded the keel and reduced the wet surface area by about half. Finally, the yaw speed was improved.

In energy harvesting, the solar panels were too small to saturate enough electricity to cross the North Atlantic. So, they considered using a 50 W micro-turbine to harvest wind energy in their next work. In addition, the sloop design was the widely used Balestron or AeroRig, also known as a balanced rig, which reduced power consumption in SOA and W2H. Furthermore, the power consumption can be reduced through a worm screw mechanical design when the motor was not working. Finally, they reduced the frequency of sensor sampling and operations for saving more energy.

The United States Naval Academy (USNA) team started collaboration with the Aberystwyth University group in building the Autonomous Robot for Rapid Transit and Ocean Observation (ARRTOO) in Figure 2 (K-6) (Miller et al., 2014). It was a sailing robot with a retractable keel and two masts each with one reef sail. It permitted high-speed motoring by reducing wind age and drag. The average power was budgeted at around 4.2 W, and the total maximum regenerative power source was 380 W, including a 260 W solar panel and 120 W wind turbine, both in peak power. During the test, the total average regenerative power source was 68 W, including a 26 W solar panel and 42 W wind turbine in average. The detailed power configuration can be found in Miller et al. (2014).

The latest sailboat collaborated with Aberystwyth University was MaxiMOOP in Figure 2 (K-7) (Miller et al., 2015). This was a small sailing vessel that can be launched and retrieved by one person. Four prototypes (Morwyn, Dewi, Mid Life Crisis, and ABoat Time) were designed and tested in short course racing and long endurance in all-weather situations, with a boat speed of around 3 knots and 2.4 knots, respectively. Additionally, they developed two control systems. One was low-energy consumption with operating power 1 W. The other higher power had complex computation. Dewi sailed a 6 h long triangular course in the 2013 SailBot competition, with the 15–20 knots wind speed and 50–75 cm wave height. The upwind speed achieved about 1 knot, and the downwind speed was between 1.5-2 knots. ABoat Time attempted the Microtransat Challenge in 2014. It sailed 220 nautical miles and evidenced 35 knots wind before it was caught by the net. After that, the MaxiMOOP was updated and widely used in the SailBot competition (MaxiMOOP, 2017).

### 3.12 UBC Sailbot, Canada

The UBC Sailbot team (Sailbot, 2016) is an engineering design team at the University of British Columbia. Initially, the team focused on the design, construction, system integration, and test of small autonomous sailboats. In 2006, they participated in the first SailBot competition. From 2009 to 2014, they built 2-m boats named Thunderbird in Figure 2 (L-1) with abilities to automatically adjust the angle of heading and sail. They won the SailBot competition via their Thunderbird sailboat in 2012, 2013, and 2014. After these achievements, they started focusing on greater challenges with large sailboats.

The first large sailboat, named Ada in Figure 2 (L-2), was a 5.5-m autonomous sailboat. It was powered by a wind sail with a uniquely canoe shape, which was designed to protect itself from the harsh weather of the North Atlantic. On August 21, 2016, Ada set a record speed in the first 3 days of sailing. But unfortunately, it encountered mechanical problems at about 700 km. The second large fully autonomous sailboat called Raye in Figure 2 (L-3), which was evolved from Ada.

### 3.13 SUPMECA and ISEN, France

Mediterranean Autonomous Robot ISEN Union SUPMECA (MARIUS) in Figure 2M (Naveau et al., 2013; Anthierens et al., 2014) was a sailboat with the whole design of Marius started from the sketch. MARIUS had to resist and sail in harsh and unknown environments in priority. To meet this requirement, the design process included robustness as a priority in mechanism, electronics, and instrument parts.

It consisted of a 2.2-m^2^ main sail and a 0.7-m^2^ jib sail. A total of two 35 W photovoltaic panels were installed on the deck like a tent. A dedicated vertical Savonius wind generator with helicoidal blades was planned with a target of 30 W energy generation. MARIUS managed its energy through three modes. Between normal and economy modes, the sampling frequency switches from 5 to 0.1 Hz for the instruments and the control of actuators. In the third mode, i.e., the critical mode, the aim was to prevent the battery from the deep discharge, and MARIUS was turned into this mode and drifted until the battery was charged above 50*%* again.

### 3.14 Instituto Universitario SIANI, Spain

ATIRMA in Figure 2 (N-1) was a 1-m class commercial carbon fiber with a main sail and foresail of Instituto Universitario SIANI (IUSIANI). The total power consumption was about 0.66 W with the battery of 22.2 Wh (3.7 V). The actuators consumed 0.6 W power with an independent battery of 19.89 Wh (7.4 V). They tested ATIRMA under an 8-h operation which consumed about 20*%* of battery capacity (Cabrera-Gámez et al., 2014).

ATIRMA G2 in Figure 2 (N-2) (Domínguez-Brito et al., 2015) was a newly designed 2-m class autonomous sailboat, which improved navigation behavior, especially in harsh conditions. In addition, it added more space for the payload and focused on robustness for sail and rudder control, with two carbon fiber wing sails and two tilted rudders. As a result, it could recover autonomously from capsizing. The twin wing sails could be used as a twin rudder in a semi-balance or compensation state, which achieved navigation successfully and improved the robustness of the rudder system. In addition, the structure helped reduce roll moments and improve performance in strong winds. The compensation structure would decrease its own torque and energy consumption.

### 3.15 Åland Univeristy, Finland

Åland Sailing Robots (ÅSR) was a project of Åland University for autonomous sailing robots. The first type of ÅSR was retrofitted from a mini 12 in Figure 2 (O-1). It was a 4-m autonomous sailboat with 8.2 m^2^ sail area and approximated 300 kg mass (Enqvist, 2016). Based on the parameters of ÅSR, the lift and drag force could be calculated. Estimating states from observations remained a huge challenge. If the problem was handled well, observations of wind and other environmental factors would save energy (Melin, 2015).

Then, Enqvist designed a symmetrical, free-rotating wing sail with a tail for mini 12 in Enqvist 2016 to meet the requirement of simplicity and reliability.

Later in 2017, Friebe’s team developed ASPire (Autonomous Sailing Platform) in Figure 2(O-3) (Friebe et al., 2017), a wind-propelled Autonomous surface vehicle (ASV) for ocean research. ASPire was a wind-propelled autonomous sailboat developed by the Åland University of Applied Sciences (Friebe et al., 2018). It was equipped with a free-rotating rigid wing sail whose power came mainly from 50 W solar panels mounted on the deck with a solar tracker. The panel was connected to a 110 Ah 12 V gel battery with 1.3 KWh of energy storage. The solar tracker operated on a single-axis to enhance energy collection. It also adopted wind-vane self-steering to steer straight against the wind in an energy efficient manner.

### 3.16 KTH Royal Institute of Technology, Sweden

Dhomé developed a 4.16-m long sailing robot Maribot Vane in Figure 2P, based on a paralympic mono-hull. A free-rotating wing sail with a flap at the tail formed an energy-efficient self-steered wind vane mechanism. Compared with the traditional sailboat rig, this was much robust and resulted in no yaw moment transferred to the hull. The limitation of this mechanism was the small delay to physically lock it after the command (Ulysse et al., 2019). The team conducted tests on the relatively protected sea near Stockholm in 3 days (Dhomé et al., 2018).

### 3.17 Shanghai Jiao Tong University, China

Wang and Xu developed a 1.5-m long monohull sailboat named SJTU Sailboat in Figure 2Q, with two triangular fabric sails in a total area of 1.152 m^2^ (Wang et al., 2015). The team started from a track-following controller, including a local path strategy, sail and rudder automatic control, and enabled autonomous sailing on the lake for verification. In Kang et al. 2016, they further applied the Velocity Made Good Method in local route planning. For long-term route planning, Du proposed a three-dimensional dynamic programming (3DDP) (Du et al., 2018) to generate a group of optimal routes with minimum voyage time and carried out a simulation for planning from Shanghai to Qingdao.

### 3.18 Cornell University, US

Students in Cornell University built the Cornell Autonomous Sailboat Team (CUSail) and developed a series of sailboats, named SailVane I, II, III, and IV in Figure 2R (Baker et al., 2015). On this basis, they proposed a monohull sailboat constructed by a weighted keel, control sail, and passive air-rudder. It allowed the sailboat to remain oriented relative to the wind without active control. Their goal was to optimize sail, keel, and air-rudder parameters and structure to achieve higher directional stability and forward speed.

Adjusting the time interval between components is a way to save energy, such as putting the system to sleep or shutting down in most cases (Baker et al., 2015). In addition, freely rotating sails and tails create angles of attack to generate lift and drag forces. In Augenstein et al. 2016, by exchanging different components in the energy-saving effect of a free rudder or angle control sail, servo rudder or passive rudder tail angle control, air-rudder or rudder tail, and air rudder or water rudder. The energy-saving group can be obtained. Finally, the sail-vane concept, which was an air-rudder–mounted downwind, seemed promising for directional and angle-of-attack stable sailing. Thus, it had potential for long-term sailing with a low electrical-energy budget. However, as shown in the high wind in 3D simulation, stability of the boat should be further improved.

### 3.19 Pierre and Marie Curie University (UPMC), France

Petres and Plumet from UPMC started robotic sailing from modeling and reactive navigation based on potential field (Petres et al., 2011), through the Autonomous SAiling Robot for Oceanographic MEasurements (ASAROME) in Figure 2S (Plumet et al., 2014) project. The 3.6-m long sailboat was based on a mini-J mono-hull, with soft main and jib sails. The battery pack was charged by a 0.5-m^2^ solar panel and a wind turbine. The solar panel was able to deliver up to 60 W under maximal lighting conditions. The wind turbine could deliver about 10 W at a wind speed of 10 knots. This regenerative energy system could deliver about 35 W on an average under typical weather conditions in Western Europe. The energy management system will activate the actuators by a rudder PD controller if heading angle is larger than 7°. Comparing with the working full-time in the embedded computers and sensors, the rudder actuator and sail actuator will be operated in 20 and 10*%* of that time, respectively. In this way, the harvested power combined with the battery pack could support 2 days of functionality. This was sufficient for the short field test, but more energy was required for long missions. As published in 2012, the preliminary field test was conducted on a river near Nantes in France.

### 3.20 University of Southampton, UK

In Lemaire et al. 2019, a 1-m Lintel monohull sailing robot called the Black Python in Figure 2T was introduced by researchers of the University of Southampton. Due to the instability of weather, wind, and waves, it was too challenging for tacking. Based on this, they proposed the method for jibing (wearing) instead, for certain situations. The Black Python was a small sailing robot for WRSC racing and did not propose much in terms of energy harvesting or management.

### 3.21 University of Florence, Italy

At the University of Florence, researchers designed a prototype sailboat called UNIFI in Figure 2U to monitor ocean areas or freshwater basins (Allotta et al., 2017). They made an ultrasonic wind sensor and calibrated in a wind tunnel (Luca et al., 2018). The energy storage system was utmost for UNIFI. Hereby, they prompted the energy-harvesting efficiency by increasing the efficient solar panel area, changing the battery packs from lead-gel to LiFePO_4_, and introducing the maximum power point tracking (MPPT) buck-boost converter (Boni et al., 2019; Boni et al., 2020).

### 3.22 The Chinese University of Hong Kong, Shenzhen, China

Researchers in the CUHK-Shenzhen adopted the more cost/time effective way by retrofitting multi-hull sport sailboats, which previously worked as human carriers and thus had a good payload capacity and durability to waves. In 2016, collaborating with the Smart China Research Institute, Hong Kong, the team developed an autonomous trimaran named Sail-Based ASV (Lam et al., 2016) in Figure 2 (V-1) retrofitted from Hobie Kayaks Adventure Island, which was 5.02 m long with one 5.47-m^2^ retractable soft sail. Due to the advantage of approximately 2.9 m width of the trimaran, a 2.6-m^2^ solar panel with peak power 440 W was equipped.

To explore whether equipping motorized propellers can save energy or not, the team developed a hybrid sailboat, named Hybrid Sailboat-II in Figure 2 (V-2), based on an around 40 cm long low-cost RC catamaran. Through the data-driven method, accurate heading control in tacking was maneuvered by motorized propellers and 23.7*%* energy saving was achieved for each loop by a 40° heading path when beating the wind (Zhang et al., 2018). Further research extended open-loop control into closed-loop PID, and about 58.9*%* energy was saved during motorized tacking (Ou et al., 2021).

In parallel with the RC catamaran, another 3.1-m long catamaran, named OceanVoy in Figure 2 (V-3), was retrofitted from an inflatable sailboat MiniCat 310. The merit of low weight enabled easy deployment for the field test. The team focused on the energy consumption optimization and proposed a hybrid energy planning method, combining the pseudo-spectral optimal control method for heading control and extreme seeking control for sail control (Sun et al., 2020). Continuous research was followed to investigate how to reduce the control frequency of the rudder, so as to decrease energy consumption and meanwhile reach the path-tracking accuracy to some extent. Based on the V-stability interval method (Jaulin and Le Bars, 2012), the team developed an E-saving approach, which was validated in field experiments of OceanVoy. The results showed that energy consumption reduced by approximately 11*%* compared to that of the previous V-stability controller (Sun et al., 2021).

## 4 Sailing Robots From Industry

A number of companies have also shown interest in sailing robots, and some successful products have been released to the market. Some of them have achieved very impressive long-term performance in ocean voyage. Most of them adopt the rigid wing sail as a propelling component. In this section, we present them with typical applications.

### 4.1 Offshore Sensing AS, Norway

The Norwegian company Offshore Sensing AS developed Sailbuoy (Sailbuoy, 2021), which was 2 m in length, equipped with a 40 W peak solar panel on the deck and 400 Wh lithium battery. One wing-sail provided propulsion force. There were two sail area choices, i.e., 0.4 m^2^ and 0.6 m^2^. In 2008, it became the first sailing robot completing the Microtransat Challenge 2018 (Microtransat, 2018). It traveled a total of 5,100 km in about 80 days to Ireland. Sailbuoy, disclosed in the specification, can provide several months sailing endurance within 3–30 m/s wind and around 15 m wave height environment.

The company also designed another robot for wave measurement, named Sailbuoy Wave. It was equipped with a wave sensor to obtain the accurate wave data. Based on the stable performance, the robot can be used in long-term monitoring missions.

Sailbuoy has been adopted as a data management platform (Langeland et al., 2019; Borge, 2015) and was widely used in the oceanographic measurements (Hole et al., 2016; Tengberg et al., 2018; Fer and Peddie, 2013; Fer and Peddie, 2016; Ghani et al., 2014), zooplankton monitoring (Pedersen et al., 2019), and region exploration. (DeYoung et al., 2020).

### 4.2 Saildrone Inc., USA

Saildrone Inc. (Saildrone, 2021a) is a company from USA focusing on oceangoing autonomous surface vehicles. Until now, the company has developed three types of sailing robots (Saildrone, 2021b): Saildrone Explorer, Saildrone Voyager, and Saildrone Surveyor with 7, 10, and 22 m in length, respectively. Their robots sailed more than 500,000 nautical miles with over 13,000 days. Solar panels were amounted on the deck and wing sails.

A large number of applications have adopted Saildrones, such as marine mammals and fishes observation (Mordy et al., 2017; Stierhoff et al., 2019; De Robertis et al., 2019; Kuhn et al., 2020), acidification observation (Tilbrook et al., 2019), cold pools observation (Wills et al., 2021), surface temperature and salinity gradients observation (Vazquez-Cuervo et al., 2020; Vazquez-Cuervo et al., 2021), ocean CO_2_ observation (Sutton et al., 2021; Marouchos et al., 2018; Sabine et al., 2020), climate observation (Meinig et al., 2019); Gentemann et al., 2020; Nagano and Ando, 2020), satellite ocean evaluation (Scott et al., 2020; Meinig et al., 2015; Cokelet et al., 2015), gas or oil seep detection (Scoulding et al., 2020; Daneshgar Asl et al., 2017), harsh environment exploration (Chiodi et al., 2021), and ice zone observation (Chiodi et al., 2021).

### 4.3 OCIUS Technology Ltd., Australia

BlueBottle (OCIUS, 2020) is a new autonomous sailing platform for ocean monitoring developed by OCIUS Technology Ltd. from Australia. It utilizes the energy from wind, solar, and wave. Solar panels on the wing sail and deck are responsible for charging the battery. The wind and wave energy are used as propulsion sources, especially when the sea state is over 6, the wing sail can be folded on the deck. The underwater flipper mechanism utilizes wave energy for propulsion.

### 4.4 Autonomous Marine Systems Inc., USA

AMS has developed Datamaran (Datamaran, 2019), i.e., a catamaran autonomous sailboat with foldable wing sail, which can help to reduce the impact from the harsh environment. The solar panels are equipped on the surface of the wing sail and the deck. They develop two-sized sailing robots: Mark 7 in 3.7 m and Mark 8 in 5 m, both with an endurance of 6 months. Some special features are in self-deployment and self-righting.

### 4.5 Ocean Aero, USA

Submaran (2021 is an autonomous sailboat from Ocean Aero. It is a 4.4-m monohull with a 200 W solar panel on the deck. This robot can fold the wing sail. In addition, it can be submerged under the surface to prevent the impact from the harsh environment.

### 4.6 Everblue Technologies Inc., Japan

Everblue (TypeA, 2020b) has designed three sailboats: Type A, Type X, and project Hydroloop. The planned applications include fish tracking, goods delivery, and hydrogen generation. The developed Type A is a 2-m long trimaran sailing robot (TypeA 2020a; TypeA, 2020c).

### 4.7 SubSeaSail, USA

The Gen6 (Gen6, 2020; Gen6, 2021), designed by SubSeaSail, has a submerged body as deep as the height of the sail on the marine surface. Solar panels are installed in the sail with an average output of around 5 W and a peak of almost 25 W. They have developed and patented a passive automatic wing control mechanism to keep the wing sail at the optimum angle for propulsion.

## 5 Robotic Sailing Competitions and Open Community

A competition has played an important role in the advancement of robotic sailing. Due to the limitations in coastal zones for testing and the difficulties in transporting sailboats, competitions are grouped into several geometric regions. Many aforementioned teams from either academia or industry have been involved. An open community has also contributed to overcome the challenge of autonomous sailing by open technologies.

### 5.1 Microtransat Challenge

The Microtransat Challenge (Microtransat, 2010) had the ambition to cross the Atlantic Ocean by autonomous sailboats. It was proposed by Dr. Mark Neal of Aberystwyth University and Dr. Yves Briere of the Institute Supérieure de l’Aéronautique et de l’Espace (ISAE) in 2005. The first competition started in 2006 on a lake. Later from 2010, there were teams started crossing the Atlantic Ocean.

Since 2015, Some teams have covered at least 1,000 km distance. The joint team by ENSTA Bretagne and Dalhousie University was the first such team, though their robot Breizh Tigresse was lost finally. Thereafter, SailBuoy, USNA, Dalhousie University, Andy Osusky, Philip Smith, Slava Asipenko, and the United States Coastguard Academy also achieved such distance. So far, only Sailbuoy successfully crossed the Atlantic in 2018.

### 5.2 SailBot

SailBot (2017) is a competition held in North America with teams from universities, colleges, and high schools. The competition supports sailing robots of up to 2 m length, also with an open sailing event of up to 4 m for non-school teams. There are five topics, including fleet racing, station keeping, endurance contest, autonomous navigation, and presentation and design.

### 5.3 World Robotic Sailing Championship

The World Robotic Sailing Championship (WRSC) (WRSC, 2019) is an international competition for autonomous sailing robots. It does not focus on ocean-crossing missions instead of promoting topics in intelligence, such as fleet race, station keeping, area scanning, and collision avoidance.

The first WRSC was held by Stelzer in Austria in 2008. Boats up to 4 m length are allowed to enter the race. WRSC rules change from year to year depending on research topics or scientific issues. The race is held in conjunction with the International Robotic Sailing Conference (IRSC). Thus, more research teams have participated and shared their thoughts and knowledge, aside from competition. It has boosted robotic sailing technology and research topics continuously.

### 5.4 Scoutbots

The group named Scoutbots (ScoutBots, 2010) focuses on developing innovative, affordable, and open technologies to collect data from the ocean. They have the motivation for detecting plastic pollution, mapping coral reef, monitoring radioactive sediments from the sea floor, sensing oil spill, etc. This group works across sectors and geographies all over the world.

They design sailboats with deployed sensors to collect data from the ocean, such as the surface or underwater robots. They build an education platform called Protei (Gernez et al., 2012), which is an open hardware shape-shifting sailing robot. So far, researchers and students have participated in the community from countries or regions for e.g., UK, Norway, Holland, Hong Kong SAR, etc.

## 6 Discussions

Numerous aforementioned research and development have provided a few numbers of valuable insights for researchers to explore long-term sailing robots from the three perspectives of energy. Actuation, the main consumer for energy, can be separated into propulsion and steering. Harvesting, the producer of electricity, seeks to earn more power resources from the environment. Energy management is to elongate working time by smartly utilizing the current electricity and enhancing energy efficiency.

### 6.1 Actuation

Propulsion takes up the major part of energy needed. Luckily, nature environmental energies are abundant on the marine surface, and sophisticated consideration in the design can strengthen the robot. Steering, another energy-consuming part in actuation, has also design tips for higher energy efficiency.

#### 6.1.1 Sail Propulsion

For the purpose of long-term sailing, maximal utilization of wind power is extremely important. For sailboats with different sizes, researchers studied and designed various sized sails. In this survey, we collected the data in sail area and sailboat length, as shown in Figure 6.

Some clues from the design can be summarized that for sailboat with length less than 4 m, the ratio of sail area over boat length empirically ranged from approximately 0.8–1.5 m^2^/*m*. But, if the boat length becomes longer, i.e., over 4 m, the ratio can exceed 2 m^2^/*m*. Researchers can take reference on the ratio to enlarge propelling force and meanwhile guarantee the motion stability.

Aside from size, an innovative sail design also attracts researchers. In the soft sail group, ASV Roboat, IBOAT, AVALON, Vaimos, SOA, and W2H are equipped with balance rigs. This structure is designed to help the sailboat keep the point of sail, which improves sail propulsion efficiency and saves energy when controlling the sail. ASV Roboat evaluates the energy efficiency of a balanced rig, saving about 68% energy. L’improbable uses a wind-vane self-steering device to adjust the trajectory of the sailboat relative to the wind. The simulation results show the effectiveness of the method.

In the wing-sail group, for e.g., Atlantis, ARC, HWT-X1, ATIRMA G2, ASPire, Saildrone, Datamaran, and Submaran, many sailboats adopt a self-trimming structure, which is a passive structure to adjust the wing sail to keep a stable point of sail at a low cost of power. Some sailboats adopt semi-balanced or compensated wing sails. Some sailboats adopt two sails to lower their plane center, with an advantage of reduced heeling moment on the hull. As a result, sailing performance in downwind and strong winds is improved.

Self-foldability, designed and demonstrated in Datamaran and Submaran, provides sailboats with robustness in large wind. This is also crucial for the long-term mission.

#### 6.1.2 Motorized Propulsion

Motorized propellers are sometimes used as auxiliary actuators, and sailboats become hybrid. In most cases, the propelling motor functions in emergencies or close-shore navigation. Research on hybrid control of motorized propeller and sails has been in the starting stage, and successful tacking due to this easy-to-control actuator can shorten the mission time and distance, but is still in the RC model sailboat level. Hybrid Sailboat II from the CUHK-Shenzhen provides the study for higher energy efficiency in a data-driven manner.

#### 6.1.3 Wave-Based Propulsion

Waves are rarely used to propel sailboats. One reason is that the wind and the waves are not pushing at the same speed. For example, the average speed of a wave glider is much slower than that of a sailboat under similar sea conditions. But, when the wind is radically strong, by folding the sail for higher safety, the robot can still achieve motion if equipped with a wave-driven mechanism. BlueBottle developed by OCIUS provides an excellent reference.

#### 6.1.4 Steering

Differing from the traditional rudder mechanism design, similar to balanced sail, ASV Roboat designs a balanced rudder to reduce the energy consumption on the rudder motor.

Another interesting idea is rudderless steering, by two wing-sails. Aberystwyth University, Cornell University, and IUSIANI explore the design. The yaw moment exerted by the masts can navigate the hull when the rudder malfunctions.

### 6.2 Energy Harvesting

Aside from energy for kinetic motions, energy for computing, sensing, and communication is also crucial for autonomy. Electricity is the main energy for such functionalities. There are two main sources to harvest electricity for sailboats: solar radiation and wind.

#### 6.2.1 Solar Energy Harvesting

From the perspective of solar energy harvesting, the area of solar panels and the efficiency caused by the assembling are two impactful points for long-term sailing.

We try to figure out the relationship between the power generation and the length of the hull. The detailed relationship diagram of collected sailboats is shown in Figure 7. It can be observed that to increase the power of solar cells, aside from increasing the boat length, adoption of multiple hulls, for e.g., catamaran or trimaran, can be very helpful, as the deck size increases significantly. The sailboats by CUHK-Shenzhen validate this, although solar cells have not fully covered the decks yet.

**FIGURE 8:**
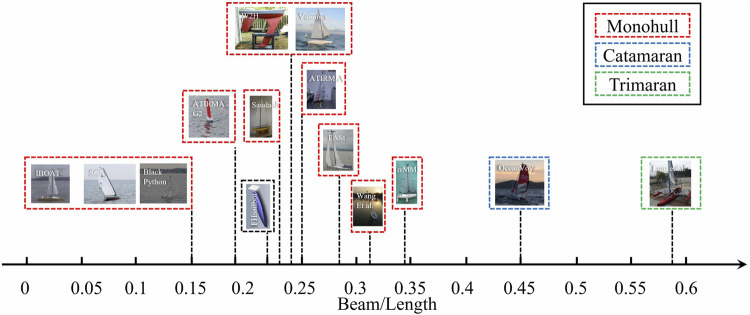
Beam-length ratio of sailboats.

To further study the deck dimension of different types of sailboats, the beam-length ratio is calculated, with details shown in Figure 8. The beam-length ratio of monohull sailboats is less than 0.35, while that of catamarans which is greater than 0.4.

The design of a house-like solar panel on the deck of Pinta has considered the direction of sunlight to enlarge the solar radiation flux into panels. In ASPire, tilted solar panels are attached to a vertical rotational axis. Interference with components on the deck and stability due to the increased height of the mass center ought to be considered in the design.

To enlarge the solar panel area, Saildrone, BlueBottle, and Datamaran Mark 8 integrate solar panels into the wing sail to capture more solar radiation. As a result, rigid wing-sail is required in such robots.

#### 6.2.2 Wind Energy Harvesting

The advantage of harvesting energy from wind is that they can generate electricity at night or on cloudy/rainy days. It functions well in downwind, but when the sailboat goes upwind, this will apparently cause drag.

Some researchers use vertical wind turbines. Compared with horizontal ones, vertical turbines can reduce wind resistance. Examples are Sailing SOA, W2H, MARIUS, ARRTOO, and ASAROME.

In addition, ENSTA’s team came up with an innovative concept, i.e., using sailboats as windmills. The sails can act as wind turbines when the boat is in downwind motion. The researchers estimated that 100 W of power would be generated in a simulated scenario.

### 6.3 Energy Management

Under the restriction of energy supply, the effective energy management scheme is one of the utmost important ways to reduce the energy consumption of electronic components while maintaining a certain sailing performance.

Researchers have proposed different energy-saving control schemes, such as rrMM’s data mining method, SJTU’s 3DDP, multimode method, ASAROME’s rudder PD controller, Aberystwyth University’s artificial endocrine controller, and OceanVoy’s E-saving method. However, effective energy management is still an open question.

#### 6.3.1 Properties in Energy Management Methods

In Table 5, some properties are described. From the “Model” column, sailboat dynamic models are merely considered in energy management approaches. One reason is that the sailboat model is difficult to build, and the simulation result cannot fit the actual experiment ideally. However, if an accurate model is available and applied, more heuristic and precise methods can be investigated.

**TABLE 5:** Comparisons among different energy management methods. “Model” indicates whether the method adopts sailboat dynamic model. “Data” represents whether the historical data is used. “Low” and “High” refer to the low-level controller and high-level controller, respectively. “Water” indicates whether wave or tide are considered. “Exp.” is used to check whether hardware experiment on the method is found. “Qua.” is used to judge whether there is quantitative data disclosed.

Boat name	Methods	Model		Data	Low	High	Water	Exp.	Qua.	Pros	Cons
rrMM	Data mining		×	*✓*	*✓*	×	×	*✓*	×	Obtain the control parameters without modeling	Sensitive to data size, robot and sailing conditions
SJTU Sailboat	3DDP		×	×	×	*✓*	×	×	×	Simulate long-term sailing	Experiment results not disclosed
SOA, MARIUS,W2H, mini 12	Multi-mode		×	*✓*	*✓*	×	×	*✓*	×	Reduce sampling or control frequency	Hardly restricted in system performance
ASAROME	Rudder PD controller		×	×	*✓*	×	×	*✓*	×	Restricted control frequency under PD control	Stability of sailing notdisclosed
Beagle-B	Artificial Endocrine controller		×	*✓*	*✓*	×	×	*✓*	*✓*	Use historical data to train without modeling the system	The expected experiment can not be achieved due to an unexplained reason
OceanVoy	E-saving method		×	*✓*	*✓*	×	×	*✓*	*✓*	Work as a lower frequency controller with an acceptable path tracking error	Disturbances such as wave and tide not considered

Despite the inexistence of the sailboat model, some methods such as data mining, multimode, artificial endocrine controllers, and E-saving methods use the historical data to fine-tune the control schemes. These methods can be implemented in their sailing robots. However, they are sensitive to different sailing robots. Hereby, historical data is needed.

The energy management controllers contain a low-level controller, a high-level controller, or both. The low-level controller works on heading or course tracking. The high-level controller focuses on path planning or waypoints generation. In Table 5, data mining, multimode, rudder PD controller, artificial endocrine controllers, and E-saving methods are applicable for the low-level control scenarios. The 3DDP method is suitable for high-level control scenarios.

In these methods, the influences of the wave or tide are merely mentioned. One reason is that the aquatic environment is not easy to model. However, the wave or tide, if studied well, can reduce the energy consumption in some situations. This metric can be considered in the future method.

In the evaluation of these methods, experimental verification shows the effectiveness and robustness of the proposed methods. Many methods in Table 5 have been implemented in real experiments. Quantitative data can be used to visually compare different methods. However, only a few methods (e.g., Beagle-B and OceanVoy) have provided such data. Hence, an open database can be established through repeatable experiments with which the research community can benefit and grow.

From the “Pros” perspective, the data mining method works based on historical sailing data without modeling, which contributes to improved navigational performance. The 3DDP method is a high-level path-planning method suitable to solve the global route planning problem. Some conclusions obtained from the simulation results of this method can be used as a reference for energy management of long-term sailing. The multimode method is a commonly adopted approach. It can reduce the sampling cycle or control frequency in sleep or idle mode to decrease energy consumption. ASAROME’s rudder PD controller can reduce the control frequency of low-level controllers. It saves energy comparing with the PD method and sails better than without the PD method. The artificial endocrine controller uses a trained model to adjust the frequency of actuator activation or sensor sampling to improve power management. The system can work without the sailboat dynamic model and obtain solutions from a trained model. The E-saving approach allows for trade-off between energy consumption and path-tracking error. It can ensure the stability of sailing.

From the “Cons” perspective, data mining approach relies on historical data, which is too sensitive to data size, sailing conditions, and different sailing robots even with the same type. The method will be more applicable if the generalization performance is improved. The 3DDP method is a high-level path-planning method. The multimode approach is too radical to avoid danger or follow its own goals in the long-sailing process. The PD method works with a low-level controller and cannot guarantee the navigation stability. The artificial endocrine controller relies on training data, and thus the quality of data is utmost important for model training. In addition, the expected experiment cannot be achieved due to an unexplained reason. The E-saving approach can be improved by combining wave and tide disturbances, so as to improve energy management performance.

As a result, most energy management approaches are based on historical data and do not consider the dynamics of sailing robots. The energy management methods are trade-off between control frequency and tracking error and require fine-tuned parameters. In addition, more effective methods can be proposed, which are able to handle both low-level and high-level control scenarios. In the future, the model and waves should be considered in energy management.

#### 6.3.2 Battery Capacity Analysis

**FIGURE 9:**
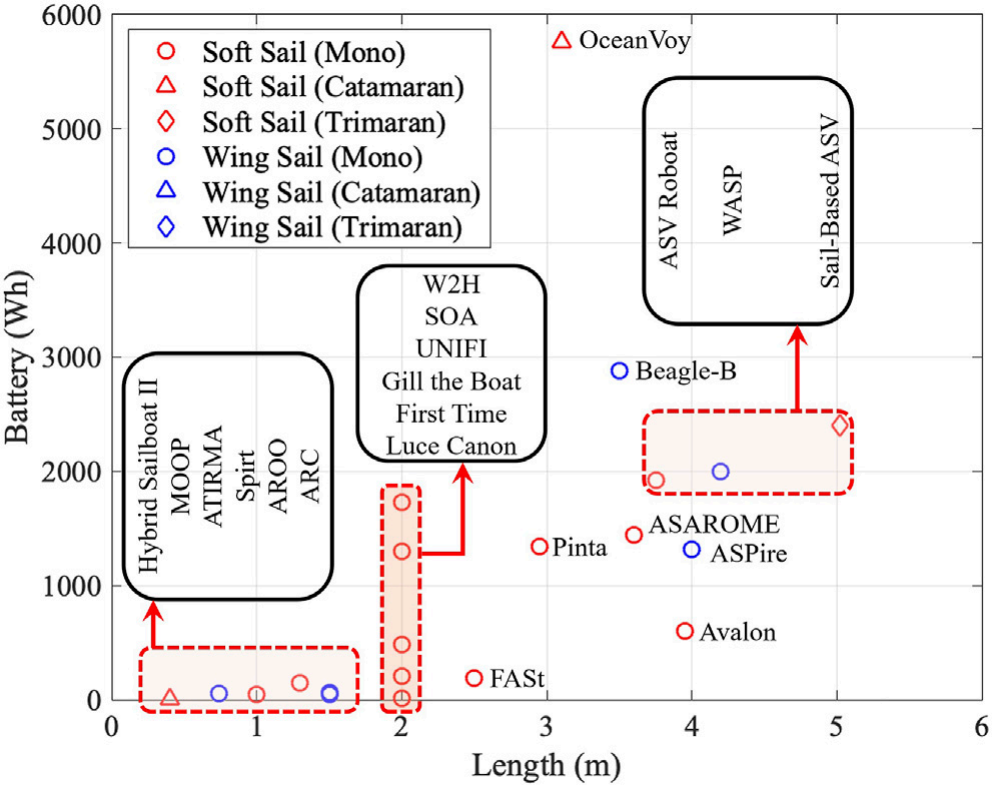
Correlation between sailboat length and battery capacity.

We analyze the relation between the length of the sailboat and battery capacity. From Figure 9, some clues can be summarized that if the sailboat is smaller than 2 m, the battery-carrying capacity is rather limited. The capacity can be empirically doubled from 1,000 Wh to around 2000 Wh with the length from 2 to 4 m approximately. Catamarans can deploy more batteries than monohull sailboats with the same length.

#### 6.3.3 Total Power Consumption Analysis

**FIGURE 10:**
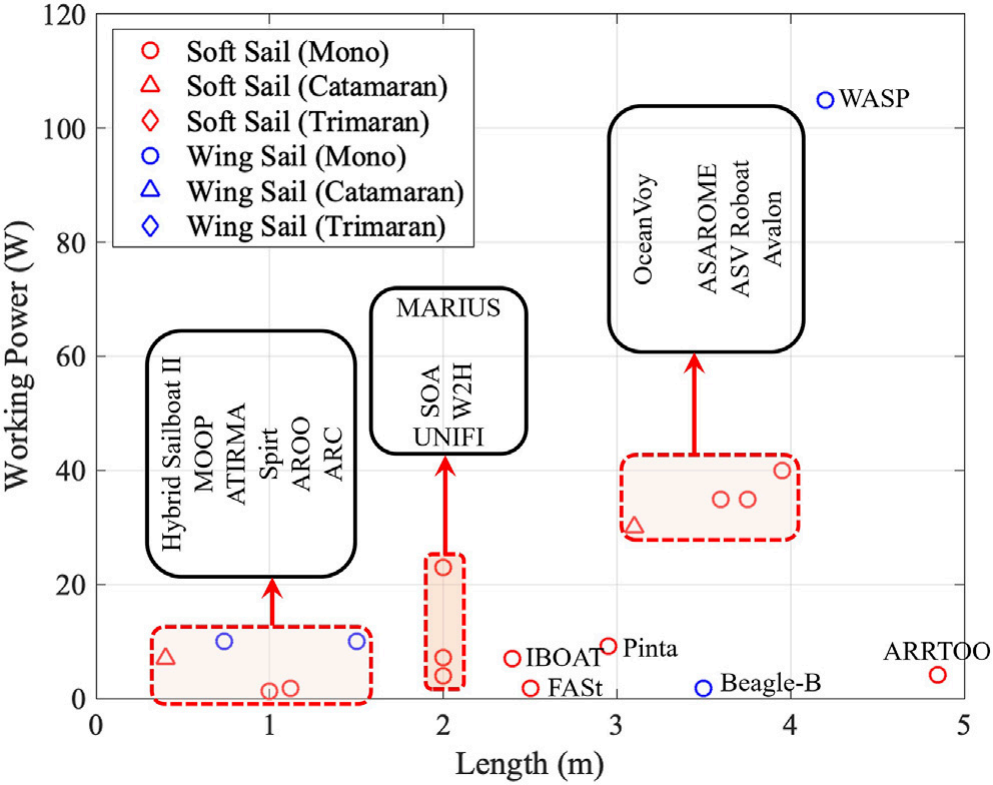
Correlation between sailboat length and working power.

The relation between the length of the sailboat and total working power data is analyzed. From Figure 10, the upper bound of operating power will be limited by the sailboat length. The results show that the large sailboat can load more working power, allowing for more sensors, computation, and more frequent actuation in rudders. The operating power range can be divided into less than 10 W, 10 W-20 W, 20 W-40 W, and greater than 40 W. From another view, even in large-sailing robots, to elongate the voyage distance, the low-working power design 10 W is welcome.

## 7 Conclusion

In this review, we investigate and summarize the existing sailing robots for the aim of long-term sailing from three perspectives in energy. Numerous efforts from academia and industry are grouped, and the research progresses in each team are elaborated. The ideas of competition and open community have also contributed to encourage and inspire research in this area. This review analyzes sailing robots with various types and dimensions of sails (soft or rigid) and hulls (monohull, catamaran, or trimaran).

To enlarge the power for propelling, i.e., the main energy expenditure, sail dimension, and the hull length shall be considered. A balanced or self-trimming sail can enhance energy efficiency. A motorized propeller has potential to save the overall power of the robot by agilely controlling the heading in tacking. Wave-based propulsion assists additionally in some urgent or harsh scenarios. For steering, another actuation aspect, a balanced rudder design can reduce energy consumption.

To harvest energy, a mainstream solution of the solar panel and its relations with hull size and type provide some clues to researchers. Solar panels can be more effective if an appropriate angle is achieved in assembly or larger area is fitted into rigid wing sail. The wind turbine and the new wind mill concept can become complimentary energy supplies.

Energy management strategies, such as the multimode method, rudder PD controller, and E-saving method. can further increase the energy efficiency. More research efforts are undergoing, and hopefully more research outcomes from the worldwide robotic society can enrich the topic of long-term robotic sailing.

The data in this survey, although are not complete, can help to provide a structural database for research studies in sailing robots to incrementally improve and lead to long-term robotic sailing.
